# Decision trees in epidemiological research

**DOI:** 10.1186/s12982-017-0064-4

**Published:** 2017-09-20

**Authors:** Ashwini Venkatasubramaniam, Julian Wolfson, Nathan Mitchell, Timothy Barnes, Meghan JaKa, Simone French

**Affiliations:** 1 Urban Big Data Centre, University of Glasgow, 7 Lilybank Gardens, Glasgow, G12 8RZ UK; 2 Division of Biostatistics, University of Minnesota, Twin Cities, A453 Mayo Building, MMC 303, 420 Delaware St SE, Minneapolis, MN 55455 USA; 3 Division of Epidemiology and Community Health, University of Minnesota, Twin Cities, West Bank Office Building, 1300 South Second St, Suite 300, Minneapolis, MN 55454 USA; 4 Division of Applied Research, Allina Health, 2925 Chicago Ave, Minneapolis, MN 55407 USA

**Keywords:** Decision trees, Subgroup heterogeneity, Predictors

## Abstract

**Background:**

In many studies, it is of interest to identify population subgroups that are relatively homogeneous with respect to an outcome. The nature of these subgroups can provide insight into effect mechanisms and suggest targets for tailored interventions. However, identifying relevant subgroups can be challenging with standard statistical methods.

**Main text:**

We review the literature on decision trees, a family of techniques for partitioning the population, on the basis of covariates, into distinct subgroups who share similar values of an outcome variable. We compare two decision tree methods, the popular Classification and Regression tree (CART) technique and the newer Conditional Inference tree (CTree) technique, assessing their performance in a simulation study and using data from the Box Lunch Study, a randomized controlled trial of a portion size intervention. Both CART and CTree identify homogeneous population subgroups and offer improved prediction accuracy relative to regression-based approaches when subgroups are truly present in the data. An important distinction between CART and CTree is that the latter uses a formal statistical hypothesis testing framework in building decision trees, which simplifies the process of identifying and interpreting the final tree model. We also introduce a novel way to visualize the subgroups defined by decision trees. Our novel graphical visualization provides a more scientifically meaningful characterization of the subgroups identified by decision trees.

**Conclusions:**

Decision trees are a useful tool for identifying homogeneous subgroups defined by combinations of individual characteristics. While all decision tree techniques generate subgroups, we advocate the use of the newer CTree technique due to its simplicity and ease of interpretation.

**Electronic supplementary material:**

The online version of this article (doi:10.1186/s12982-017-0064-4) contains supplementary material, which is available to authorized users.

## Background

The framing of medical research hypotheses and development of public health interventions often involve the identification of high-risk groups and the effects of individual factors on the relevant outcome [1, 2]. For example, the prevalence of obesity in the United States has more than doubled in the past 30 years [3, 4] and this trend can be associated with a complex combination of factors in the data. However, excessive calorie consumption and inadequate physical activity are not solely responsible for this problem; numerous other factors such as socio-economic differences, demographic characteristics, physical environment, genetics, eating behaviors, etc. also influence the energy intake balance and weight status.

While individual effects can be measured efficiently, characterizing these factors in relation to an outcome of interest can be challenging. Effects of continuous variables (e.g., age) may be non-linear, and vary with other continuous (e.g., years of education) and categorical (e.g., sex) variables. Regression models have long been utilized for prediction and to examine the relationships between covariates and responses of interest. However, their ability to identify interactions between covariates and relevant population subgroups is restricted by the data analyst’s decision about how covariates are defined and included in the model. For example, even in the very simple case of partitioning the population into two maximally distinct groups on the basis of a single continuous predictor *X*, one would need to fit separate models with categorical predictors indicating that *X* exceeded a particular threshold value, for many different threshold values. Since many candidate models may have to be investigated in this somewhat *ad hoc* manner, Type I error may be inflated.

The main goal of this paper is to introduce and describe the family of statistical methods known as decision trees, a family which is particularly well-suited to exploring potentially non-linear relationships between variables and identifying population subgroups who are homogeneous with respect to outcomes. Decision trees have been utilized to identify joint effects of air pollutants [5], generate a realistic research hypothesis for tuberculosis diagnosis [6], and recognize high-risk subgroups to aid tobacco control [7]. After providing a brief overview of decision trees, we introduce a novel data visualization technique for summarizing the subgroups identified by the trees. Next, we explore the differences between a commonly used technique for building decision trees, CART, and the conditional inference tree (CTree) approach which has not been widely used in epidemiological applications. Based on simulation results and analyses of real data, we discuss the relative strengths and weaknesses of these two approaches and the resulting implications for data analysis.

### Application: the Box Lunch Study

Throughout this paper, we present examples and analyses based on variables collected in the Box Lunch Study (BLS), a randomized controlled trial designed to evaluate the effect of portion size availability on caloric intake and weight gain in a free living sample of working adults. The main randomized comparisons of the BLS (along with details of ethics approval and consent information) have been reported elsewhere [8, 9]. However, the data also provides the opportunity to explore associations between outcomes and individual characteristics. Available covariates include demographic (e.g. age, gender, race, height, education), lifestyle (e.g. smoking status, physical activity levels), and psycho-social measures (e.g. frequency of self-weighing, degree of satisfaction with current weight). Responses to the Three Factor Eating Questionnaire (TFEQ) [10] quantifying the constructs of hunger, disinhibtion, and restraint were also recorded. The BLS also collected data on some novel, laboratory-based psycho-social measures that had not previously been measured in a randomized trial setting such as the relative reinforcement of food (rrvf), liking and wanting.

### Software availability

The analyses, simulations, and visualizations presented in this paper were all produced using the freely-available statistical software R [11–14]. External packages and functions used are referenced in the text. Code for our novel visualization is available at https://github.com/AshwiniKV/visTree and for reproducing our example trees and our simulation study at https://github.com/AshwiniKV/obesity_decision_trees.

## Methods

### A brief introduction to decision trees

A decision tree is a statistical model for predicting an outcome on the basis of covariates. The model implies a *prediction rule* defining disjoint subsets of the data, i.e., population subgroups that are defined hierarchically via a sequence of binary partitions of the data. The set of hierarchical binary partitions can be represented as a tree, hence the name. The predicted outcome in each subset is determined by averaging the outcomes of the individuals in the subset. The goal is to create a prediction rule (i.e., a tree) which minimizes a *loss function* that measures the discrepancy between the predicted and true values.

**Fig. 1:**
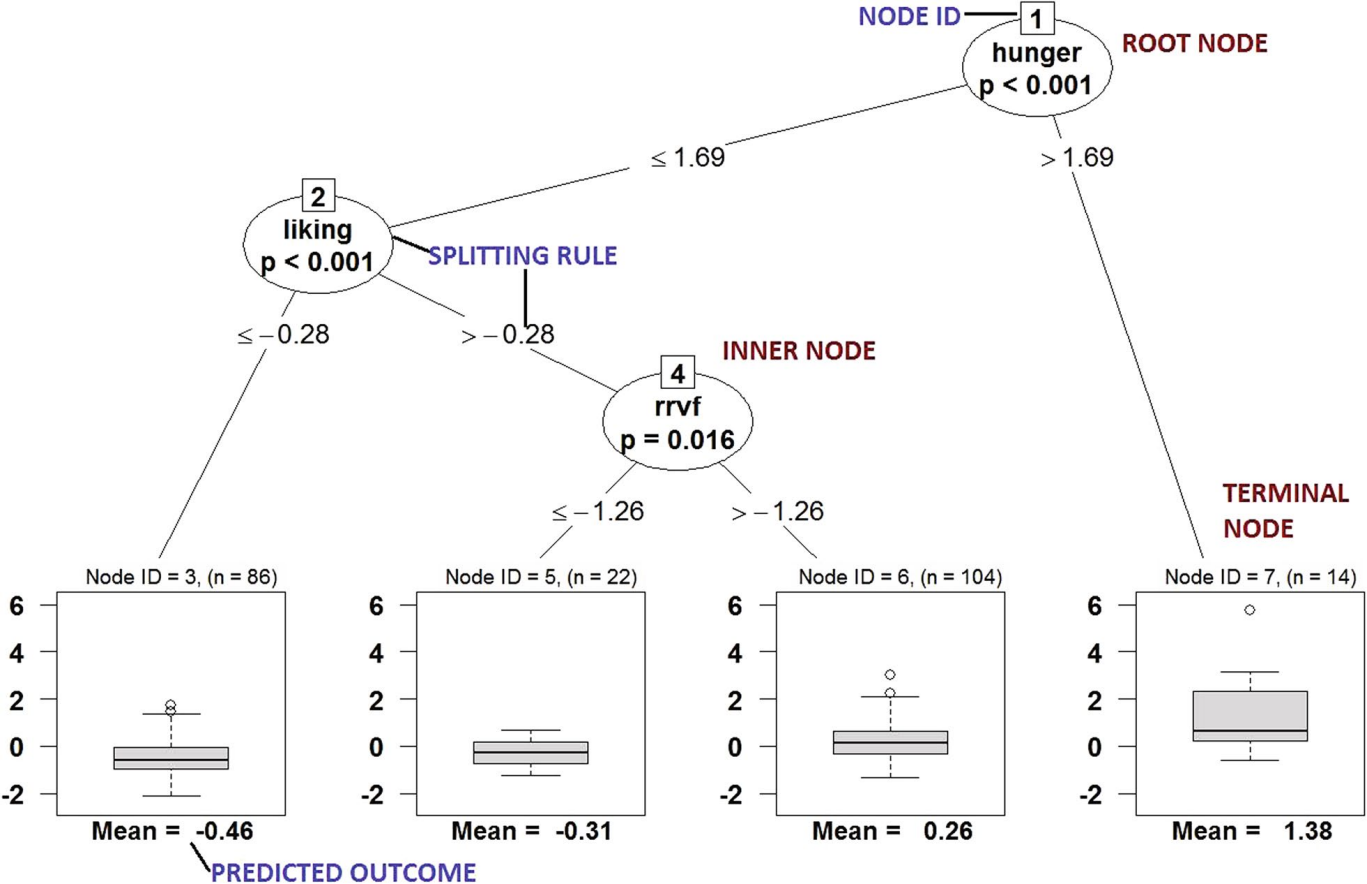
Decision tree showing the association between daily caloric intake (in kcal/day) and hunger, dis-inhibition, restrained eating, relative reinforcement, liking, and wanting. All measures are obtained at baseline in the Box Lunch Study

Decision trees have several components, as illustrated in Fig. 1 which summarizes the association between the outcome of daily caloric intake and hunger, dis-inhibition, restrained eating, relative reinforcement, liking, and wanting. *Nodes* contain subsets of the observations; the *root node* of a tree (labeled with a ‘1’ in Fig. 1) contains all observations ($n=226$ in the Box Lunch Study). The key step in algorithms for constructing decision trees is the *splitting step*, where the decision is made on how to partition the sample (or sub-sample, for nodes below the root) into two disjoint subsets according to covariate values. The splits below a node are represented as *branches* in the tree. Splitting continues recursively down each branch until a *stopping rule* is triggered. A node where the stopping rule is satisfied is referred to as a *leaf* or a *terminal node*. Taken together, the terminal nodes define a disjoint partition of the original sample; each observation belongs to exactly one terminal node, depending on its covariates. A prediction for a new observation’s outcome is made by determining (based on that observation’s covariates) which leaf it belongs to, then combining the outcomes of the existing observations within that leaf to get a predicted value.

In Fig. 1, both the outcome and predictors are standardized column-wise to have mean zero and variance equal to one. Standardization puts all the predictors on the same scale, which may be helpful when, as here, some of the predictors (e.g., rrvf, liking, and wanting) are measures that do not have universally agreed-upon units or methods of measurement^1^. For example, in Fig. 1, the root node with a label ‘1’ as node ID partitions the population into two groups: (1) subjects whose hunger measurement is less than or equal to 1.69 standard deviations above the mean hunger, and (2) subjects whose hunger is greater than 1.69 standard deviations above the mean. Standardizing the outcome allows for a similar interpretation of the leaf nodes: the leaf with node ID = 6 has a value of 0.26, indicating that the mean 24-h energy intake for the subjects contained in this node (i.e., those with hunger ${\le }1.69$, liking ${>}-0.28$, and rrvf ${>}-1.26$) is 0.26 standard deviations above the overall mean of 24-h energy intake. A mean of 0.26 standard deviations of 24-h energy intake corresponds to a value of 2190 kilo-calories^2^.

### Adjusting for covariates

Often, factors such as age, sex, and education level may influence the outcome of interest and be associated with other predictors (i.e., they are confounders), but their effects are not of primary interest. In linear regression, it is common practice to adjust for such variables by including them in the regression model.

In decision trees, an analogue to covariate adjustment involves building the tree using *adjusted residuals*, i.e., residuals from a regression model containing the confounders. To be precise, suppose that one wished to assess the effects of the predictors described in the previous sections, adjusting for age, sex, and BMI. Letting *Y* denote 24-h energy intake, one would first fit the model

1 $$Y = \beta _0 + \beta _1 \ {\hbox {Age}} + \beta _2 \ {\hbox {Sex}} + \beta _3 \ {\hbox {BMI}} + \epsilon$$

Given coefficient estimates ${\hat{\beta }}_0, {\hat{\beta }}_1, {\hat{\beta }}_2$, and ${\hat{\beta }}_3$, the age-, sex, and BMI-adjusted residuals for 24-h energy intake, $Y^*$, are

2 $$Y^* = Y - {\hat{\beta }}_0 - {\hat{\beta }}_1 \ {\hbox {Age}} - \hat{\beta }_2 \ {\hbox {Sex}} - {\hat{\beta }}_3 \ {\hbox {BMI}}$$

The residuals $Y^*$ can then be used as the outcome in a regression tree including the predictors of interest. This adjusted residuals technique can be easily applied using standard software.

### Visualizing subgroups in decision trees

**Fig. 2:**
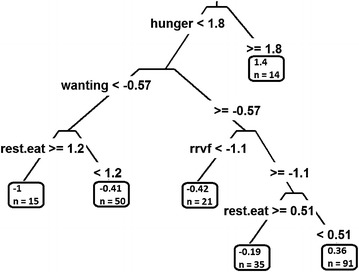
Regression tree showing the association between Energy kcal/day and hunger, dis-inhibition, restrained eating, relative reinforcement of food, liking, and wanting

One of the most attractive features of decision trees is that they partition a population sample into subgroups with distinct means. However, the typical display of a decision tree (e.g., Figs. 1 and 2) does not always allow researchers to easily characterize these subgroups. The problem is particularly acute if some of the predictor variables do not have an interpretable scale built on established norms: the relative reinforcing value of food and degree of liking/wanting measured in the Box Lunch Study are novel and have not yet been widely used, so a standard unit of measurement has not yet been established.

**Fig. 3:**
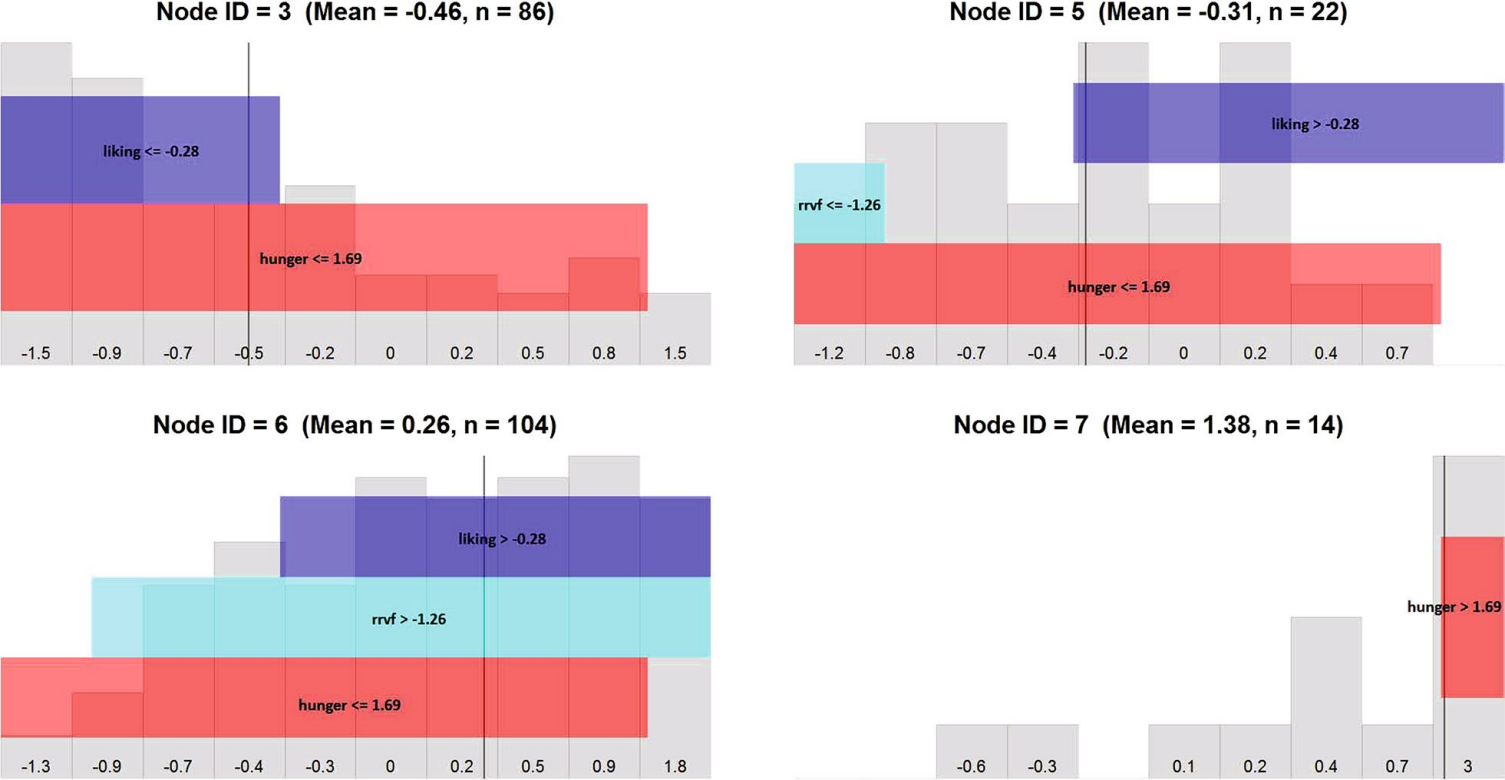
Graphical visualization of the conditional inference tree in Fig. 1, where the visualization consists of a grid of plots and each plot corresponds to a terminal node

To address this limitation, we developed a software tool for visualizing the composition of subgroups defined by decision trees. The visualization consists of a grid of plots, one corresponding to each terminal node (i.e., population subgroup). In Fig. 3, each plot in this grid of plots corresponds to one of the four terminal nodes (population subgroups) in Fig. 1, i.e. nodes 3, 5, 6, and 7. In the background of each plot is a histogram summarizing the distribution of the outcome variable (here, 24-h energy intake) for the individuals in the terminal node/subgroup. For example, the top left plot in Fig. 3 shows a distribution of (standardized) 24-h energy intake that is right-skewed. The numbers along the x-axis are the average 24-h energy intake within each individual bin of the histogram. The mean of the values contained in the bins of the histogram are presented for each individual bin. The vertical line shows the overall mean of the subgroup; the mean and subgroup size are shown in the plot title. Overlaid on the background are colored bars; the length and position of the bars represent the set of predictor values, on the percentile scale, which define the subgroup. The subgroup corresponding to the top left plot of Fig. 3 is defined by liking values below −0.28, which represents the 39th population percentile and hunger values that are below 1.69, which represents the 91st percentile.

This visualization summarizes, at a glance, the characteristics of the groups determined by the regression tree. For instance, in Fig. 3, the four groups could be characterized as:

- *Group 1 *($N = 86$): Moderate to low liking, all but very high hunger. This group has below-average energy intake (standardized mean = −0.46).
- *Group 2 *($N = 22$): Moderate to high liking, very low relative reinforcing value of food, all but very high hunger. This group has moderate to low energy intake.
- *Group 3 *($N = 104$): Moderate to high liking, all but very low relative reinforcing value of food, all but very high hunger. This group has moderate to high energy intake.
- *Group 4 *($N = 14$): Very high hunger. This group has very high energy intake.

The prediction rules defining these subgroups provide insight into the individual characteristics that can affect the outcome, and can be used to define categorical variables that could yield more meaningful and interpretable comparisons in future analyses.

### Methods for building decision trees

#### Classification and regression trees (CART)

The most popular method for constructing decision trees, known as CART (Classification and Regression Trees) was introduced by Breiman [18]. In a CART (e.g., Fig. 2), a split is sought to minimize the *relative sum of squared errors* in the two partitions resulting from the split. The search for splits in CART takes place across two dimensions simultaneously: the covariate to split on and splitting point within that covariate. In other words, the splitting step in CART is greedy: the best split is sought across all covariates and candidate split points for those covariates. For binary and categorical covariates, all possible values are considered as possible split points; for continuous covariates, an equally-spaced grid covering the range of possible values is usually considered.

Because it searches over all possible splits on all covariates, CART is vulnerable to the so-called *biased variable selection problem*; there are more potential “good” splits on a continuous-valued covariate (or one with a large number of distinct values) than on a binary covariate. This tendency of CART to favor variables with many possible splits has been described in [18–20] and [21].

Furthermore, the nature of the splitting process makes it difficult to describe the statistical properties of any particular split. For instance, CART is not concerned with the notion of Type I error since it does not control the rate at which a regression tree identifies population subgroups when there is truly no heterogeneity in the mean of the outcome.

#### Conditional inference trees (CTree)

As an alternative to CART, Hothorn et al. [22] proposed the conditional inference tree (CTree). Unlike CART, CTree (e.g., Fig. 1) separates the splitting process into two distinct steps. The first step is to determine the variable to split on based on a measure of association between each covariate and the outcome of interest. Then, after the splitting variable has been determined, the best split point for that variable is calculated.

In contrast to CART, CTree follows formal statistical inference procedures in each splitting step. The association between each covariate and the outcome is quantified using the coefficient in a regression model (linear regression for continuous outcomes and other suitable regression models for other outcome types), and a node is only chosen to be split if there is sufficient evidence to reject the *global null hypothesis*, i.e., the hypothesis that none of the covariates has a univariate association with the outcome. If the global null hypothesis is rejected, then the covariate that displays the strongest association with the outcome of interest is selected as a candidate for splitting. If the minimum p-value is larger than the multiplicity adjusted significance threshold, then no variable is selected for splitting and the node is declared a terminal node. Note that, despite its name, CTree bases splitting decisions on marginal (i.e., univariate) regression models; the “conditional” refers to the fact that, following the initial split, subsequent inference takes place within subgroups, i.e., conditional on subgroup membership.

#### Stopping rules

In both CART and CTree, splitting continues until a *stopping rule* triggers. In CART, splitting stops when the relative reduction in error resulting from the best split falls below a pre-specified threshold known as the *complexity parameter*. Typical values of this parameter are in the range of 0.001–0.05. To prevent overfitting, it is common practice to construct trees for a sequence of values of this parameter, and select the final value by minimizing prediction error estimated by cross-validation or on an independent test set. This process is referred to as *pruning* [23, 24]. A slightly more conservative stopping rule sets the final complexity parameter to the value which yields a prediction error one standard deviation larger than the minimum estimated by cross-validation or on an independent test set. This is known as the *1-SE rule*. As noted above, CTree’s stopping rule is simple: splitting stops if the global null hypothesis is not rejected at the pre-determined, multiplicity adjusted level of significance.

### Comparing CART and CTree: a simulation study

In this section, we describe simulated and real data and develop scenarios within a simulation study to highlight distinctions between CART and CTree. We also compare their predictive performance to standard regression models in a variety of settings and perform simulations utilizing the R statistical software package, version 3.3.0 [11]. The results of this study are presented in “Results” section. The CART algorithm was implemented using the rpart package [13], while the CTree was implemented via the partykit package [12]. We considered a variety of scenarios where we varied the data-generating function, covariate type (categorical vs. continuous), the sparsity (proportion of variables predicting the outcome), the total sample size, and the complexity parameter for CART.

For all scenarios other than the one where sample size was varied, the sample size was fixed at 250 and in all scenarios trees were constructed using six covariates. Continuous outcomes were generated as independent $N(\eta ,1)$ with linear predictor $\eta$ varying across scenarios as described below. Continuous covariates were generated from independent Normal distributions with mean zero and unit variance; binary covariates were generated as independent Bernoulli($p = 0.5$). Pruning for CART was carried out using both the minimum and the 1-SE rule, with the 1-SE rule being implemented using the DMwR package [14]. The tree-generating functions rpart (for CART) and ctree (for CTrees) were applied with arguments specifying a minimum of 20 observations for a node to be considered for splitting and a minimum of 7 observations in a terminal node. The complexity parameter for CART was held at the default value of 0.01. The level of significance in the CTree was held at the default value of $\alpha = 0.05$.

For each scenario, 10,000 simulations were performed, where in each simulation a training dataset was simulated and used to construct the trees, and tree performance was evaluated on an independently generated test dataset. Prediction error and tree complexity were summarized respectively via the mean squared error (MSE) and the number of terminal nodes (equal to the total number of splits in the tree, plus one).

#### Effect of the data generating process

Decision trees perform well in situations where the underlying population is partitioned into a relatively small number of subgroups with distinct means. However, they are less suited to scenarios in which the outcome varies continuously with covariate values.

We started by generating independent normally distributed outcomes according to a pre-specified tree structure, i.e., set of splits to seven terminal nodes with mean values (−1.88, −0.30, −0.31, 0.25, −0.09, 2.23, 1.35), and unit variance. The candidate covariates for this tree included six continuous covariates ($X_1, \dots , X_6$), mimicking the six covariates considered in the introductory examples above. This CTree is grown to consist of seven terminal nodes with splits at hunger, liking, rrvf, and disinhibition.

In a different scenario, continuous responses are generated from $N(\eta ,1)$ where $\eta$ follows a regression model defined as

$$\eta = 1.5 X_1 + 1.25 X_2 + 1 X_3 + 0.85 X_4 + 0.75 X_5 + 0 X_6$$

and $X_1 \dots X_6$ are simulated as independent normally distributed continuous covariates. We also generated a hybrid model from normally distributed data with unit variance according to $N(\eta ,1)$ with

$$\begin{aligned} \eta &\,= \,0.5 X_1 + 0.45 X_2 + 0.3 X_3 + 1.5\,{\mathbb{1}}(X_1 \le 0, X_2> 0, X_3 \le 0) +\\&\qquad 0.25\,{\mathbb{1}}(X_1 \le 0, X_3 >0) + 0.14\,{\mathbb{1}} ( X_1> 0, X_2>  0), \end{aligned}$$

where $X_1, X_2$, and $X_3$ are simulated as independent normally distributed continuous covariates and are utilized to form distinct subgroups represented by three different indicator functions, indicated by $\mathbf 1$. This hybrid model includes main effects of three continuous covariates along with interaction terms and subgroup indicators constructed from these covariates.

#### Type I error

We also evaluated the Type I error rate of the different tree-building algorithms. For a tree, we say that a Type I error occurs if a tree splits on a variable that has no association with the outcome. To evaluate Type I error, we generated six independent and normally distributed continuous covariates and a response with mean zero and unit variance, unrelated to the covariates.

#### Effect of sample size

**Fig. 4:**
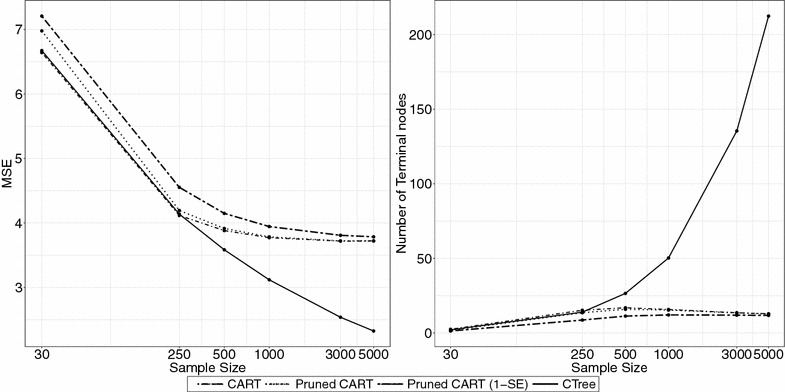
Prediction error and tree size for different sample sizes in log-scale ($n = 30, 250, 500, 1000, 3000, 5000$) when data is generated from a linear regression model with continuous covariates

Figure 4 summarize the predictive performance of tree types as sample size changes. For each sample size $n = 30, 250, 500, 1000, 3000$, and 5000 we generated six covariates and continuous responses were generated from a N($\eta , 1$) with $\eta$ following a linear regression model:

$$\eta = 1.5 X_1 + 1.25 X_2 + 1 X_3 + 0.85 X_4 + 0.75 X_5 + 0 X_6.$$

## Results

### Comparing CART and CTree: a simulation study

#### Effect of the data generating process

The set of *Tree* results for the model that generates data from a tree structure in the first five rows of Table 1 summarizes the estimated prediction error (MSE) and tree complexity (mean, 20th, and 80th percentile number of terminal nodes) of CTree on the generated data with a comparison to three other tree algorithms: the unpruned CART, CART with two types of pruning, and with the results from a linear regression model. As expected, all the tree-based techniques have lower MSE than linear regression. In this case, CTree produces trees with a similar number of terminal nodes to the CART pruned with the 1-SE rule but lower number of nodes when compared to the regular pruned CART. The CTree and both types of pruned CARTs have results for decision trees with 3–4 terminal nodes, in contrast to the generated tree structure with seven terminal nodes. This is likely due to the fact that our simulated tree data contained several nodes with very similar means.

**Table 1:** Aggregated simulation results that describe the effect of multiple types of data generating processes

True model	Type	MSE		Terminal nodes		
		Mean	SD	Mean	20th	80th
Tree	CART	1.26	0.151	7.01	6	8
	Pruned CART	1.22	0.137	4.27	3	5
	Pruned CART (1-SE)	1.25	0.139	3.31	3	4
	CTree	1.27	0.154	3.72	3	4
	Linear regression	2.04	0.179			
Regression	CART	4.12	0.413	15.24	14	16
	Pruned CART	4.19	0.442	13.97	12	16
	Pruned CART (1-SE)	4.55	0.509	8.66	6	11
	CTree	4.14	0.409	13.96	13	15
	Linear regression	1.03	0.093			
Hybrid	CART	1.39	0.138	13.1	11	15
	Pruned CART	1.37	0.131	5.96	3	9
	Pruned CART (1-SE)	1.39	0.133	2.69	2	3
	CTree	1.34	0.126	5.42	4	6
	Linear regression	1.17	0.106			

These sources of data include a tree structure, a regression model and a hybrid model that combines the two structures

The second set of results in Table 1 (*Regression*) summarize performance for all four model types. The (correctly specified) linear regression model has far better predictive performance than the tree models. Interestingly, CTree has better predictive accuracy than the pruned versions of CART, a result which agrees with the findings of Schaffer [25] that pruning does not necessarily improve predictive accuracy, particularly when there are many (here, infinitely many) subgroups.

For the *hybrid* scenario when data is generated from the defined hybrid model, we compare the performance of the trees to a partially misspecified linear regression model containing only the main effect terms for the continuous covariates and the results in Table 1 show that predictive accuracies are relatively similar.

#### Type I error

The results are presented in Table 2. We found that the unpruned CART algorithm continues to split and grow unlike the pruned CARTs and CTree. CARTs pruned using a 1-SE rule are rather conservative with a very low Type I error while the pruned CART and CTree have Type I errors that are closer to 0.05. As noted below, explicit control of the Type I error rate is an advantage of the CTree approach.

**Table 2:** Aggregated results of simulations that evaluate Type I error of different tree building algorithms

Type	MSE		Type I error
	Mean	SD	Mean
CART	0.65	0.07	1
Pruned CART	0.99	0.091	0.0559
Pruned CART (1-SE)	1	0.089	0.0003
CTree	0.99	0.089	0.0513
Linear regression	0.97	0.088	

#### Effect of sample size

We observe in Fig. 4 that as sample size increases, the MSE of CTree continues to improve while that of the CART variants levels off beyond $n=500$. The reason for this behavior is that CART’s stopping rules are based on a complexity parameter, which sets a lower bound for improvement in model fit which is insensitive to sample size. In the rpart package, the default complexity parameter value is 0.01, so splitting stops if no split improves model fit by at least 1%. In this setting, the covariates have continuous linear effects, which implies an infinite number of population subgroups. Hence, most splits will yield small improvements in model fit, and CART variants will “stop too soon” and have poor predictive performance. In contrast, the stopping criterion for the CTree is based on *p* values, and maintaining a fixed *p* value threshold with increasing sample size allows splits associated with smaller and smaller effect sizes to be represented in the tree.

### Application

We illustrate the application of decision trees to the Box Lunch Study by comparing a linear regression model and decision tree that seek to predict 24-h energy intake (in kcal/day) using a set of 25 covariates measured at baseline. These prediction models were built on the covariates introduced in “Application: the Box Lunch Study” section such as restrained eating, rrvf, liking as well as other covariates that record demographic characteristics including age, sex, and BMI. Other covariates included were psycho-social measures such as “Influence of weight on ability to judge personal self”, “Ability to limit food intake to control weight (days/month)”, and “Frequency of weighing oneself”.

To provide a baseline for comparison, we present results from a linear regression model in Table 3. The covariates listed are those selected using backward elimination with the AIC. While there are many significant covariates in Table 3, this linear regression does not provide any information about potential interactions nor does it identify particular population subgroups that share similar values of the outcome.

**Table 3:** Linear regression output for modeling 24-h energy intake using a “suitable” set of predictors

	Estimate	SE	*t* value	Pr(${>}|\hbox {t}|$)
(Intercept)	1279.36	211.78	6.04	<0.001***
Sex: male	378.03	66.30	5.70	<0.001***
Body mass index	16.68	6.96	2.40	0.017*
Snack-energy kcal/day	1.29	0.12	10.76	<0.001***
Fruit/vegetable svg/day	38.84	14.94	2.60	0.010**
Sugar-sweetened beverage svg/day	114.20	30.3234	3.77	<0.001***
Contour drawing rating scale-body dissatisfaction [1–9]	−48.44	26.2195	−1.85	0.066
Frequency of self-weigh				
Never	(Ref)			
About once a year or less	−405.34	145.47	−2.79	0.006**
Every couple of months	−247.32	137.55	−1.80	0.074
Every month	−374.43	147.96	−2.53	0.012*
Every week	−414.77	138.67	−2.99	0.003**
Every day	−450.17	166.89	−2.70	0.008**
Fast food frequency				
Never	(Ref)			
1–3 times last month	14.13	77.01	0.18	0.855
1–2 times per week	35.63	95.42	0.37	0.709
3–4 times per week	−187.55	204.63	−0.92	0.360
5–6 times per week	−235.81	237.61	−0.99	0.322
7 or more times per week	738.04	238.35	3.10	0.002**
Hunger	32.52	10.15	3.20	0.002**
Wanting	2.88	0.85	3.40	<0.001***

This “suitable” set of predictors is chosen using a backward elimination process, such that the AIC for the relevant model is minimized

**Fig. 5:**
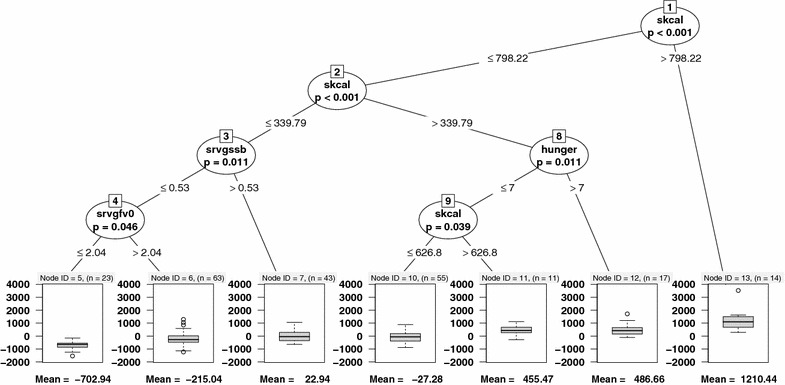
Conditional inference tree representing the relationship between adjusted residuals for daily energy intake (adjusted for age, sex, and BMI) and 22 baseline covariates. Added Node ID labels in the terminal node. This is consistent with the titles for each subplot in Fig. 3 and the CTree in Fig. 1

Figure 5 shows a conditional inference tree to predict total energy intake, adjusted for age, sex, and BMI, from 22 baseline covariates. The corresponding CART regression tree is provided in Additional file 1. The overall structure and splitting of the CART and CTree are similar, though CART has more splits than CTree. The prediction mean-squared error (using scaled energy intake values) for the conditional inference tree in Fig. 5 is 0.67 compared to 0.48 for the linear regression in Table 3. While the mean squared error is lower for linear regression, it may provide only limited scientific insight into the complex mechanisms underlying energy intake. Only the decision tree enables the identification of meaningful population subgroups and allows for formal inference about the defined groupings. For example, at the top level of the tree, the variable most strongly associated with (adjusted) total energy intake is snack calories (skcal, $p < 0.001$). Splitting the population according to snack calories $\le$798.22 versus >798.22 produces two subgroups. Within the first group (following the left branch in Fig. 5), snack calories remain the most significant predictor of total energy intake ($p < 0.001$), while in the second group (the right branch of Fig. 5) none of the covariates are significantly associated with the outcome. The first group (skcal $\le$798.22) again splits into two groups: snacking calories $\le$339.79 and >339.79 (but $\le$798.22). In the former, “low snacking” group, the covariate most strongly associated with total energy intake is servings of sugar-sweetened beverages (srvgssb, $p = 0.01$), which defines subgroups according to whether individuals consumed $\le$ or >0.53 SSBs per day. In the latter, the strongest association is with hunger ($p = 0.01$), which splits into subgroups according to *hunger* $\le$7 or >7. The lower hunger group splits one more time on snack calories. Within the former “low snacking” group that splits to define a subgroup that consumes $\le$0.53 SSBs per day, the covariate most strongly associated with energy intake is servings of fruits and vegetables (srvgfv0, $p = 0.044$), which defines subgroups according to whether individuals consumed $\le$ or >2.04 servings per day.

In general, decision trees are typically used to describe the associations between a set of covariates and an outcome, and thereby identify population subgroups with different outcome values. In our setup, there is no one particular exposure or treatment variable of interest, so there is not one focal variable whose effect may be modified by others. However, recursive partitioning does identify relevant interactions between covariates, i.e., combinations of covariate values which result in different (mean) values of the outcome. Hence, if the term “effect modification” is identified with “interaction”, then decision trees can be viewed as a tool for exploring effect modification.

**Fig. 6:**
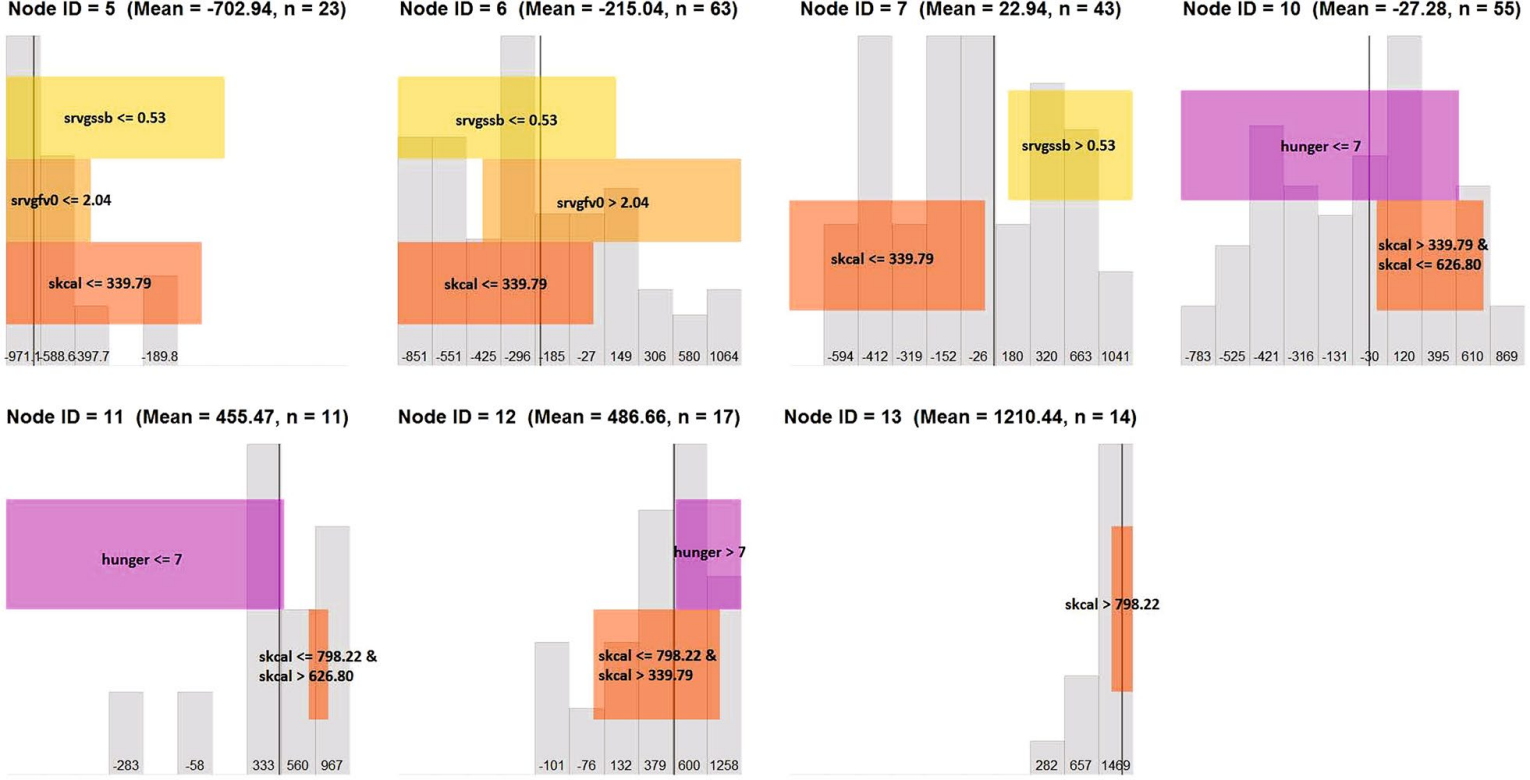
Graphical visualization to display the composition of the 7 subgroups defined by the tree in Fig. 5

Figure 6 is composed of 7 sub-plots that represent each of the terminal nodes (i.e., subgroups) in Fig. 5. The top left sub-plot in Fig. 6 corresponds to node #5 ($n = 23$) in Fig. 5. The mean of adjusted residuals is −702.94, indicating that on average, individuals in this node have a daily energy intake 702.94 kcal lower than the age-, sex-, and BMI-adjusted population mean. In the top left sub-plot in Fig. 6, colored horizontal bars describe the population subgroup of node #5: individuals with low to moderate servings per day of sugar-sweetened beverages ($\le$0.53 servings per day, i.e., below the 60th population percentile), low servings per day of fruits and vegetables ($\le$2.04 servings per day, i.e., below the 25th population percentile) and low to moderate snack calories ($\le$339.79 kcal per day, below the 50th population percentile).

The bottom row of plots corresponds to the three nodes which had the highest adjusted average caloric intake (+455.47, +486.66, and +1210.44 kcal/day relative to the adjusted population mean, respectively). These nodes defined three distinct subgroups: (1) low to moderate hunger ($\le$7, below the 80th percentile) and relatively high snacking (627–798 kcal/day, between the 89th and 92nd percentiles); (2) high hunger (>7, above the 80th percentile) and moderate snacking (340–798 kcal/day, between the 58th and 92nd percentiles); and (3) very high snacking calories ($\ge$ 798 kcal/day, above the 92nd percentile). The fact that the first two of these groups have relatively similar adjusted mean daily caloric intake while being defined by distinct combinations of hunger and snacking levels (low hunger, moderate to high snacking in the first group vs. high hunger, moderate snacking in the second) suggests that there are multiple pathways which lead to similar levels of consumption of excess calories. These distinct pathways may require different intervention strategies: for example, the low hunger but moderate to high snacking group might be effectively targeted by an approach which sought to reduce snacking opportunities, under the logic that due to their relatively low hunger level they are more likely to be snacking out of convenience than to satisfy a craving. The high hunger but more moderate snacking group, on the other hand, might be more responsive to an approach aimed at managing cravings. Yet another approach might be required to optimize outcomes for the third group whose extremely high adjusted daily caloric intake (+1210.44 kcal/day relative to the population) was associated with extremely high snacking but not hunger.

## Conclusions

Decision trees can be a powerful tool in a researcher’s data analysis toolbox, providing a way to identify relevant population subgroups which may provide insight into associations and effect mechanisms, and suggest strategies for tailoring interventions. In this paper, we compared two techniques for constructing decision trees, CART and CTree, and introduced a novel graphical visualization technique for decision trees which allows a researcher to see and compare the characteristics of these subgroups. Our focus was on describing relationships between a relatively small number of continuous or binary covariates and continuous outcomes in studies with moderate sample sizes, but decision trees can easily be extended to problems with larger sample sizes [26, 27], greater number of covariates, and for modeling other covariate and outcome types [28, 29]. The CTree approach in particular accommodates a wide variety of data types, including categorical and time-to-event outcomes, within the same statistical framework.

While the data we used to illustrate the application of decision trees arose from a randomized controlled trial, we performed cross-sectional analyses on baseline data and hence did not use information on treatment assignment. As with any technique based on identifying statistical associations, decision tree methods do not estimate causal effects of individual characteristics or exposures in such cross-section analyses. The adjustment procedure we describe above allows the researcher to account for measured variables that are thought to be confounders, but the additional flexibility provided by decision tree models cannot correct for bias due to unmeasured confounding. Hence, conclusions based on decision tree analysis should be viewed as exploratory. In ongoing work, we are extending the decision tree framework to characterize (causal) treatment effect heterogeneity (i.e., causal effect modification) in the context of randomized intervention studies.

The two decision tree fitting techniques we compared in this paper, CART and CTree have different strengths and weaknesses. CART has the advantage of availability: it is widely implemented in standard statistical software packages, while to our knowledge, conditional inference trees are currently only implemented in R. In our experiments, CART often had slightly higher predictive accuracy than CTree due to its additional flexibility. However, CTree offers several advantages over CART. First, CTree yields a simpler tree building process as compared to CART, since in CTree a single overall Type I error rate parameter ($\alpha$) controls the size of the tree and removes the need for pruning. The $\alpha$ value can be set independent of the outcome type (e.g., continuous, binary, time to event, etc.), unlike for CART where the complexity parameter depends on the splitting criterion which may differ depending on the outcome type. By using formal inferential techniques incorporating multiplicity adjustments to select splits, CTree provides statistical guarantees and valid *p* values at each split. Hence, the researcher deciding which technique to use must consider the relative value of giving up a small amount of model flexibility and predictive accuracy to simplify modeling and gain the ability to make formal statistical statements based on the results from the fitted tree.

## Additional file

**Additional file 1.** Regression tree representing the relationship between adjusted residuals for energy intake (adjusted for age, sex, and BMI) and 22 baseline covariates

## Abbreviations

CART: classification and regression trees
CTree: conditional inference tree
BLS: Box Lunch Study
BMI: body mass index
MSE: mean squared error
SE: standard error
TFEQ: three factor eating questionnaire

## Footnotes

1 Some studies record participant’s self-reported level of wanting and liking using quantitative scales (e.g., [15]), while other studies measure this via brain activity during a motivational state (e.g., [16, 17]).

2 This standardized value of 0.26 is calculated from $(2190 - 2012)/685.55$, where 2012 is the mean energy intake and 685.55 is its standard deviation.
