# Supplementary figures and images for: Decision trees in epidemiological research
Source: Emerg Themes Epidemiol. 2017 Sep 20;14:11. doi: 10.1186/s12982-017-0064-4 (PMC5607590; doi:10.1186/s12982-017-0064-4)

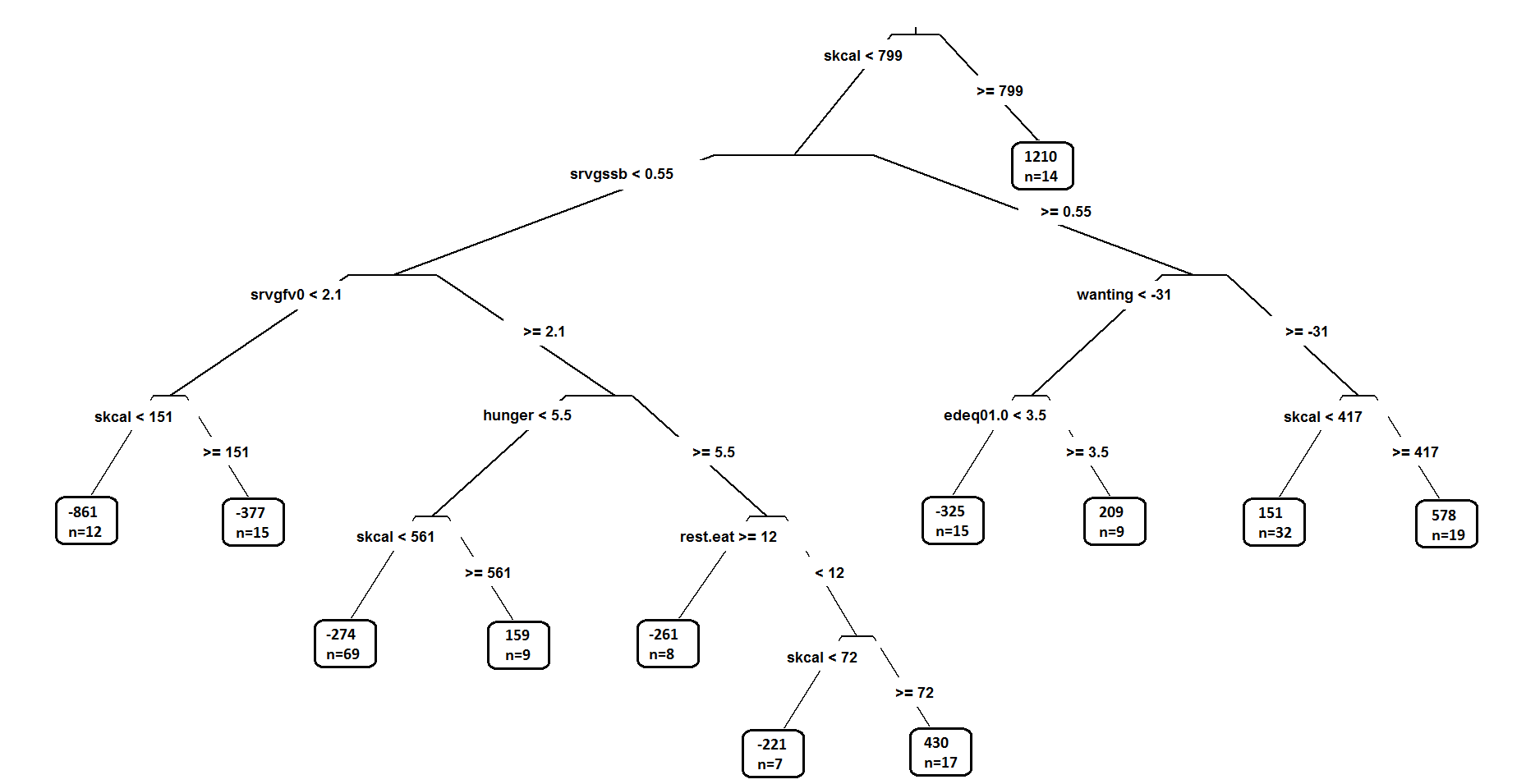

Supplement: Supplementary file 1 — Additional file 1. Regression tree representing the relationship between adjusted residuals for energy intake (adjusted for age, sex, and BMI) and 22 baseline covariates [file 12982_2017_64_MOESM1_ESM.png]
